# Changes in physical activity during the retirement transition: a series of novel n-of-1 natural experiments

**DOI:** 10.1186/s12966-017-0623-7

**Published:** 2017-12-08

**Authors:** Suzanne McDonald, Rute Vieira, Alan Godfrey, Nicola O’Brien, Martin White, Falko F. Sniehotta

**Affiliations:** 1 Institute of Health & Society, Newcastle University, Baddiley-Clark Building, Richardson Road, Newcastle upon Tyne, NE2 4AX UK; 2 Department of Computer and Information Science, Northumbria University, Newcastle upon Tyne, NE2 1XE UK; 3 MRC Epidemiology Unit, University of Cambridge School of Clinical Medicine, Box 285 Institute of Metabolic Science, Cambridge Biomedical Campus, Cambridge, CB2 0QQ UK; 4 Fuse, UKCRC Centre for Translational Research in Public Health, Newcastle University, Newcastle uponTyne, NE2 4AX UK

**Keywords:** N-of-1 methods, Physical activity, Retirement, Older adults

## Abstract

**Background:**

Existing evidence about the impact of retirement on physical activity (PA) has primarily focused on the ***average*** change in PA level after retirement in group-based studies. It is unclear whether findings regarding the direction of PA change after retirement from group-based studies apply to individuals. This study aimed to explore changes in PA, PA determinants and their inter-relationships during the retirement transition at the individual level.

**Methods:**

A series of n-of-1 natural experiments were conducted with seven individuals who were aged 55–76 years and approaching retirement. PA was measured by tri-axial accelerometry. Twice-daily self-report and ecological momentary assessments of evidence- and theory-based determinants of PA (e.g. sleep length/quality, happiness, tiredness, stress, time pressure, pain, intention, perceived behavioural control, priority, goal conflict and goal facilitation) were collected via a questionnaire for a period of between 3 and 7 months, which included time before and after the participant’s retirement date. A personalised PA determinant was also identified by each participant and measured daily for the duration of the study. Dynamic regression models for discrete time binary data were used to analyse data for each individual participant.

**Results:**

Two participants showed a statistically significant increase in the probability of engaging in PA bouts after retirement and two participants showed a significant time trend for a decrease and increase in PA bouts over time during the pre- to post-retirement period, respectively. There was no statistically significant change in PA after retirement for the remaining participants. Most of the daily questionnaire variables were significantly associated with PA for one or more participants but there were no consistent pattern of PA predictors across participants. For some participants, the relationship between questionnaire variables and PA changed from pre- to post-retirement.

**Conclusions:**

The findings from this study demonstrate the impact of retirement on individual PA trajectories. Using n-of-1 methods can provide information about unique patterns and determinants of individual behaviour over time, which has been obscured in previous research. N-of-1 methods can be used as a tool to inform personalised PA interventions for individuals within the retirement transition.

**Electronic supplementary material:**

The online version of this article (10.1186/s12966-017-0623-7) contains supplementary material, which is available to authorized users.

## Background

Physical activity (PA) is an independent risk factor for several chronic diseases such as diabetes, cardiovascular disease and cancer [1]. Therefore, it is recommended that adults engage in at least 150 min of moderate-vigorous PA per week, accumulated by engaging in bouts lasting at least 10 min in duration [1]. These PA guidelines apply to adults of all ages. However, a large proportion of adults in the UK, aged 60 years and over, do not meet these PA levels [2]. Encouraging older adults to increase PA is a key public health challenge.

The retirement transition may represent an opportunity to promote healthier behaviours and habits that persist into older adulthood [3–5]. Previous studies have shown that PA levels change in response to retirement but findings are inconsistent regarding the direction and magnitude of PA change, with some studies showing that PA levels increase [6, 7] and others showing PA levels decrease [8, 9] after retirement. Pre-retirement occupation type (i.e. manual vs. non-manual) has been suggested as a potential moderator [10, 11]. In other words, individuals retiring from physically demanding occupations may experience a net decrease in PA, whereas those retiring from sedentary occupations may experience changes in the opposite direction after leaving work. However, recent studies do not support this hypothesis [12].

Several other factors may determine the magnitude and direction of PA change after retirement, but only a limited number of potential PA determinants have been studied [13, 14]. Findings from qualitative research show that individuals approaching retirement and those who have recently retired believe that a wide range of factors influence their PA after retirement [13, 15]. For example, individuals report that there is more time availability, energy and motivation for engaging in PA after retirement, and new social roles and opportunities arise, which may either facilitate or conflict with engaging in PA [15]. Retirement has also been associated with changes in sleep length/quality, mood, fatigue and stress [16–18], which are factors commonly reported as determinants of PA [19]. PA levels and determinants are perceived to be dynamic in nature and may change several times during the first few years of retirement [15, 20].

Understanding changes in PA levels and determinants during the retirement transition is essential for designing effective interventions targeting this period. Due to the multiple inter-related changes that may take place during the retirement transition, individual PA trajectories may vary considerably. However, most existing evidence about PA change after retirement has been derived from longitudinal or cross-sectional studies that tend to focus on the ***average*** change in PA, obscuring individual PA trajectories. Furthermore, longitudinal studies typically involve few (self-reported) measurements of PA, which provides limited information about PA change over time [14, 21]. Research methods that capture intra-individual change and variability in PA, and determinants thereof, may add a crucial perspective.

N-of-1 methods involve the repeated measurement of an individual over time allowing conclusions to be drawn about the individual. They are emerging as a viable research method in health behaviour research [22, 23] and have been previously applied to study PA [24, 25]. The aim of this study was to use n-of-1 methods to explore whether PA, PA determinants and their inter-relationships change during the retirement transition within individuals.

## Method

### Design

A series of n-of-1 natural experiments were conducted in participants’ natural environment. Retirement represented a naturally occurring ‘intervention’. Data were collected from participants on a daily basis for a minimum of 2 months (up to a maximum of 7 months) covering a period pre- and post-retirement (at least 1 month pre- and 1 month post-retirement). The length of the study period was based on participant willingness to extend participation beyond the minimum 2-month period.

### Participants

Individuals who were in full- or part-time employment and had a planned date of retirement within the subsequent 6 months were eligible to participate. Participants were recruited via study advertisements placed in the community and sent to charity organisations in Newcastle upon Tyne, UK. Although retirement was self-defined by the participant, individuals retiring from full-time to part-time employment were eligible to participate only if working hours were reduced by at least 50%. The study was approved by Newcastle University’s Medical Sciences Ethics Committee (00630_1/2013).

### Measures

#### Initial meeting

Each participant completed a demographic questionnaire. Participants’ home postcodes were collected and entered into the database from the Office for National Statistics website (www.ons.gov.uk) to obtain an index of multiple deprivation (IMD) score [26], which was used as an indicator of socio-economic status. Typical levels of occupation-related PA over the previous year were collected via the EPIC PA questionnaire [27]. A semi-structured interview was conducted with each participant to elicit views about anticipated PA change after retirement and the determinants of their own PA. Responses were used to formulate personalised questionnaire items for inclusion in the daily measures.

#### Daily measures

PA data were collected at 60-min epochs during a period pre- to post-retirement using wrist-worn PRO-Diary devices (CamNtech, Ltd.; Cambridge, UK) designed to collect objectively-measured tri-axial accelerometry data and self-reported questionnaire data. During the study period, participants were prompted to complete a brief questionnaire displayed on the PRO-Diary in the morning and evening. Two audible prompts occurred at a time selected by each participant during their initial meeting with the researcher. Participants then responded to questions on a visual analogue scale with anchors appropriate to each item. The morning questionnaire included 10 items; the first two items collected data about the number of hours and quality of sleep the participant had the previous night. The remaining items were ecological momentary assessments (EMA) of happiness, tiredness, stress, time pressure, pain, intention, perceived behavioural control (PBC) and priority to engage in PA. The evening questionnaire included two items that measured whether other daily activities or goals had conflicted with or facilitated engaging in PA [28] and a personalised item, which was formulated at the initial meeting (see Additional file 1 for questionnaire items). Participants were asked an additional three questions in the morning and an additional two questions in the evening that were related to sedentary behaviour and are not reported here.

### Procedure

#### Initial meeting

Participants provided written consent, completed questionnaires and received a demonstration of the PRO-Diary. PA ***bouts*** were the study outcome of interest. PA was defined to participants as any type of PA that lasted at least 10 consecutive minutes.

#### Data collection period

Participants were contacted via email/telephone each week to check for potential problems and met a researcher face-to-face on a monthly basis to receive a fully-charged PRO-Diary for the subsequent month of data collection. Participants did not receive feedback about their data during the study. Daily data were collected for a minimum of 2 months. Participants were invited to extend their participation (up to a maximum of six additional months). Depending on the participant’s retirement date, this involved further data collection in the pre- and/or post-retirement period. Participants received a £10 ($12.44) gift voucher at each monthly meeting to reimburse participation.

#### Post-study feedback

Participants were given personalised oral and written feedback about their PA over the study period (e.g. PA levels and PA predictors). The participant’s graphed data were used to stimulate discussion and interpretations with the participant. A semi-structured interview was conducted to elicit views about the acceptability of participating in an n-of-1 study (data are not presented here).

### Data analyses

#### Data processing

Minute-by-minute PA data were downloaded from the PRO-Diary device and combined into one file for each participant. The number of PA bouts performed per day was calculated using an automated algorithm (available upon request from the authors). At the initial meeting, participants chose on which wrist they would prefer to wear the PRO-Diary. The participant wore the device on the same wrist for the duration of the study. PA data were interpreted based on cut-points which defined when the participant was performing PA of any intensity; ≥215 raw counts was used when the device was worn on the left (non-dominant) wrist and ≥385 raw counts was used for the right (dominant) wrist, based on a comparable association [29] to a wrist-worn device utilised in a previous study [30]. These cut-points are equivalent to ≥1.5 METS. To distinguish sleeping and awake periods, awakening was identified with five consecutive minutes of non-zero counts after 04:00 h. Sleeping was identified when there were two hours with 0 raw counts, interrupted with a maximum of three minutes of non-zero counts. As participant 7 wore the PRO-Diary overnight, sleeping was identified when there were two hours with 0 raw count, interrupted with a maximum of 10 non-consecutive minutes of non-zero counts. Missing PA data was identified when there was a string of 0 raw counts interrupted by a maximum of two minutes of PA within a 60-min period. If the participant removed the PRO-Diary for the day, sleeping time for those days was defined as the median sleeping time of all other days. Data from the moment the PRO-Diary was removed to sleeping time were considered missing data. Missing questionnaire data were identified and imputed using the average value of the three previous and three subsequent days.

#### Statistical analysis

PA was considered as longitudinal binary data (i.e. PA or no PA) measured each minute during the study period. Dynamic regression models [31] for discrete time binary data were used to express and model each participant’s PA behaviour over time. Time *(t)* was assumed to be a discrete variable such that *t* = 0,1,… T, where T is the total number of minutes of awake time for the participant. The observations of PA bouts (Y) form a subject-specific binary process Y_1_,…,Y_T_, where Y_t_ = 1 if the participant initiates a PA bout at minute *t,* Y_t_ = 0 otherwise.

The dynamic regression modelling procedure used in this study has been described in detail elsewhere [32]. Briefly, two sets of covariates, X_t_ and D_t,_ which vary over time are considered. The first, X_t_, describes exogenous variables such as trend over time, day of the week (weekend, workday), period of the day (morning: waking time-12:00, afternoon: 12:00–18:00, evening: 18:00-sleep time; with afternoon used as the reference variable) together with endogenous variables such as retirement period (pre-retirement, post-retirement) and predictor variables from the morning and evening questionnaire (scale: 0.0–1.0). The second, D_t,_ are dynamic covariates constructed to summarise the history up to *t* of responses Y_1_,…,Y_t_ for the individual. The number of PA bouts in the previous two hours was selected to account for autocorrelation (i.e. dependency between the data points due to repeated measurements from the same individual) between consecutive PA bouts while the number of PA bouts *d* days earlier for *d* = 1,2 adjusts for the effect of the amount of exercise done in the two previous days. Other dynamic variables included questionnaire variables for one (lag 1) and 2 days prior (lag 2). Independence of data points is assumed, as the conditional distributions given earlier responses are modelled.

Descriptive, univariate and multivariate analyses were performed. As the number of potential predictors was large and it was of interest to determine the most influential variables, a stepwise strategy was used to select the best fitting multivariate model according to Akaike’s Information Criterion. Retirement period, as well as time, weekday, period of day, number of bouts in last 2 h and number of bouts in the previous day/two days were forced into the model regardless of significance for each participant to control for any existing time trend, periodicity or autocorrelation. Potential interactions between retirement period and other exogenous/endogenous variables were explored by including the interaction terms in the stepwise process. Global goodness-of-fit was assessed with the Hosmer and Lemeshow test [33]. Multicollinearity was assessed using variation inflation factors for the predictors included in the final model. Data were analysed for each participant individually using R 3.2.3. The significance level was set at *p* < 0.05.

## Results

### Participant characteristics

Table 1 displays participant characteristics. Seven right-handed individuals (five female) aged 55–76 years who were approaching retirement responded to advertisements and provided written informed consent to participate. Only one participant (participant 7) wore the PRO-Diary on the right (dominant) wrist, therefore, higher cut-point values were used for this participant [30]. All participants reported their pre-retirement occupation within the ‘sedentary’ classification on the EPIC physical activity questionnaire [27], except participant 2 who reported their occupation within the ‘standing’ classification. All participants agreed to extend their participation beyond the initial 2-month study period and provided daily data for between 3 and 7 months. The total number of study days ranged from 87 (participant 6), to 196 (participants 3 and 4) days.

**Table 1:** Characteristics of the seven participants

Participant	Sex	Age	Marital status	Occupation^a^	Retirement	IMD^b^ score
1	Female	65	Married	Librarian (FT)	Full retirement	10
2	Female	76	Widowed	Shop owner (PT)	Full retirement	7
3	Male	56	Married	Civil servant (FT)	Full retirement	6
4	Male	60	Married	HR manager (FT)	Full retirement	9
5	Female	64	Married	Secretary (PT)	Full retirement	3
6	Female	55	Married	Nurse (FT)	Full retirement	10
7	Female	63	Divorced	Nurse (FT)	Part time work^c^	9

^a^FT = full time (≥ 35 h per week); PT = part time (<35 h per week)

^b^IMD = Index of multiple deprivation

^c^Working hours reduced from 37.5 per week to 16 h per week

### Descriptive statistics

Participant 5 had the highest amount of missing (non-wear) PA data during awake periods (4.2%) while participant 1 wore the accelerometer throughout all awake periods. The median amount of hours awake per day for each participant ranged from 13.6 to 16.3. A method of data imputation was not used for PA data since missing PA data were minimal (Additional file 2 displays further details about awake/non-wear time per participant).

The median number of daily PA bouts ranged from 4 to 13 in the pre-retirement phase and from 5 to 15 in the post-retirement phase (see Table 2). Participant 2 experienced the highest decrease in the median number of PA bouts per day (pre-retirement: 12, post-retirement: 9) while participant 3 experienced the highest increase (pre-retirement: 13, post-retirement: 15). The median number of minutes spent in a PA bout ranged from 13 to 15 in the pre-retirement phase and 13 to 17 in the post-retirement phase. Participant 1 did the least minutes of PA per day, (median: pre-retirement 57 min; post-retirement 74 min) while participant 3 did the most (median: pre-retirement 239 min; post-retirement 303 min).

**Table 2:** Descriptive statistics for the number of PA bouts and number of minutes in PA bouts for each participant pre- and post-retirement

No. of PA bouts								No. of minutes in PA bouts	
	All day	Morning		Afternoon		Evening			
Participant	Per day^a^	Per day^a^	Total^b^	Per day^a^	Total^b^	Per day^a^	Total^b^	Per bout^a^	Per day^a^
1									
Pre	4 (1,12)	1 (0,6)	66 (32.8)	2 (0,7)	93 (46.3)	1 (0,6)	42 (20.9)	13 (10,51)	57 (12,213)
Post	5 (1,14)	1 (0,8)	159 (38.2)	2 (0,10)	196 (47.1)	0 (0,4)	61 (14.7)	13 (10,99)	74 (10,279)
2									
Pre	12 (3,24)	2 (0,6)	403 (36.0)	4 (0,10)	522 (46.6)	1 (0,6)	196 (17.5)	15 (10,81)	204 (50,431)
Post	9 (3,21)	5 (1,9)	357 (41.1)	5 (1,11)	365 (42.1)	2 (0,6)	146 (16.8)	14 (10,71)	146 (34,348)
3									
Pre	13 (3,24)	4 (1,11)	385 (38.8)	5 (0,12)	366 (36.9)	3 (0,7)	240 (24.2)	15 (10,124)	239 (42,514)
Post	15 (3,26)	6 (0,11)	671 (40.7)	5 (0,11)	656 (39.8)	3 (0,7)	323 (19.6)	17 (10,133)	303 (67,571)
4									
Pre	7 (2,16)	2 (0,7)	244 (35.3)	3 (0,10)	308 (44.6)	1 (0,6)	139 (20.1)	15 (10,176)	127 (31,330)
Post	8 (3,18)	4 (0,8)	383 (41.9)	4 (0,10)	411 (45.0)	1 (0,5)	120 (13.1)	15 (10,150)	145 (37,411)
5									
Pre	11 (3,19)	4 (0,9)	168 (33.6)	4 (1,9)	201 (40.2)	3 (0,8)	131 (26.2)	15 (10,90)	197 (68,480)
Post	10.5 (3,21)	3 (0,9)	249 (29.4)	5 (0,10)	389 (45.9)	2 (0,10)	210 (24.8)	15 (10,100)	184 (40,426)
6									
Pre	8 (2,14)	2 (0,7)	83 (31.8)	3 (0,9)	117 (44.8)	2 (0,5)	61 (23.4)	15 (10,85)	131 (29,262)
Post	9 (2,16)	2 (0,6)	121 (24.6)	5 (0,10)	237 (48.3)	2 (0,10)	133 (27.1)	15 (10,108)	173 (31,463)
7									
Pre	7.5 (4,19)	3 (0,9)	120 (39.6)	3 (0,9)	124 (40.9)	1 (0,6)	59 (19.5)	14 (10,48)	126 (55,296)
Post	7 (0,20)	2 (0,7)	372 (40.0)	3 (0,12)	409 (42.9)	1 (0,5)	173 (18.1)	13 (10,60)	101 (0,365)

^a^Reporting: median (range)

^b^Reporting: n (%)

Missing questionnaire data ranged from 3.4 to 8.6%. Visual inspection of the data for each participant indicated that missing data did not occur systematically (e.g. according to the day of the week). Tables 3 and 4 summarise daily questionnaire data. The median length of self-reported sleep for pre-retirement and post-retirement ranged between 6 and 8.5 h. The median number of hours of sleep pre- and post-retirement appeared to be similar for most participants, with 0.5 h increase and decrease for participants 6 and 1, respectively. In relation to sleep quality, participant 6 had the lowest median scores (pre-retirement: 0.4; post-retirement: 0.6) while participant 3 had the highest median scores (pre-retirement: 1.0; post-retirement: 1.0). The median sleep quality score increased post-retirement for all participants except participant 3 and 7. The median score for happiness, PA intention and PA facilitation increased or stayed the same post-retirement for all participants, whereas the median score for stress, time pressure and PA conflict decreased or stayed the same post-retirement for all participants. The median score for tiredness decreased post-retirement, except for participants 5 and 7, where the median tiredness score increased, and for participant 3, where the median tiredness score stayed the same. For all participants the median PA PBC score decreased post-retirement and the median PA priority score increased post-retirement. For participants 1, 4, 6 and 7 the median score on their personalised item decreased post-retirement, whereas for participant 5 it increased and for participants 2 and 3 it stayed the same post-retirement.

**Table 3:** Descriptive statistics for the morning questionnaire data for each participant pre- and post-retirement^a^

Participant	Sleep length	Sleep quality	Happiness	Tiredness	Stress	Time pressure	Pain	PA intention	PBC	PA priority
1										
Pre	7.5 (3.5,9.5)	0.7 (0.1,0.9)	0.8 (0.4,0.9)	0.4 (0.1, 0.8)	0.2 (0.0,0.9)	0.3 (0.0,0.8)	0.7 (0.3,0.8)	0.6 (0.3,0.8)	0.5 (0.2,0.8)	0.4 (0.3,0.9)
Post	7.0 (5.0,9.0)	0.8 (0.6,0.9)	0.8 (0.5,1.0)	0.3 (0.1,0.8)	0.2 (0.0,0.6)	0.2 (0.0,0.6)	0.6 (0.5,0.8)	0.7 (0.3,0.8)	0.3 (0.1,0.8)	0.5 (0.3,0.7)
2										
Pre	8.5 (7.0,9.5)	0.8 (9.3,1.0)	0.7 (0.1,0.9)	0.3 (0.1,0.6)	0.4 (0.1,0.8)	0.3 (0.0,0.8)	0.0 (0.0,0.5)	0.6 (0.2,0.9)	0.5 (0.2,0.8)	0.6 (0.3,0.8)
Post	8.5 (5.5,10.5)	0.9 (0.3,0.9)	0.8 (0.1,0.9)	0.2 (0.1,1.0)	0.2 (0.1,0.7)	0.2 (0.0,0.8)	0.0 (0.0,0.9)	0.7 (0.1,0.9)	0.2 (0.1,0.9)	0.7 (0.2,0.9
3										
Pre	6.0 (3.0,7.5)	1.0 (0.0, 1.0)	1.0 (0.1,1.0)	0.4 (0.0,1.0)	0.0 (0.0,0.9)	0.0 (0.0,1.0)	0.0 (0.0,0.9)	0.4 (0.0,1.0)	0.8 (0.0,1.0)	0.3 (0.0,1.0)
Post	6.0 (3.0,7.5)	1.0 (0.0, 1.0)	1.0 (0.2,1.0)	0.4 (0.0,1.0)	0.0 (0.0,0.8)	0.0 (0.0,1.0)	0.0 (0.0,0.8)	0.6 (0.5,1.0)	0.5 (0.0,1.0)	0.6 (0.0,1.0)
4										
Pre	7.0 (4.0,9.0)	0.7 (0.2,0.9)	0.8 (0.3,0.9)	0.7 (0.1,1.0)	0.2 (0.0,0.7)	0.6 (0.0,0.8)	0.1 (0.0,0.7)	0.6 (0.1,1.0)	0.6 (0.0,0.9)	0.3 (0.1,1.0)
Post	7.0 (3.5,8.5)	0.8 (0.0,1.0)	0.9 (0.6,1.0)	0.3 (0.0,0.9)	0.1 (0.0,1.0)	0.2 (0.0,0.9)	0.2 (0.0,0.8)	0.6 (0.1,0.9)	0.2 (0.1,0.9)	0.5 (0.1,0.9)
5										
Pre	6.0 (5.0,7.0)	0.8 (0.4,0.9)	1.0 (0.1,1.0)	0.1 (0.0,0.6)	0.1 (0.0,0.8)	0.6 (0.0,0.9)	0.0 (0.0,0.0)	0.4 (0.1,0.9)	0.8 (0.2,0.9)	0.3 (0.1,0.8)
Post	6.0 (5.0,7.0)	0.9 (0.2,1.0)	1.0 (0.5,1.0)	0.3 (0.0,0.9)	0.0 (0.0,0.1)	0.0 (0.0,0.8)	0.1 (0.0,0.3)	0.7 (0.2,0.9)	0.4 (0.1,1.0)	0.6 (0.1,0.8)
6										
Pre	7.0 (5.0,8.0)	0.4 (0.0,1.0)	0.6 (0.2,0.8)	0.8 (0.2,1.0)	0.5 (0.2,1.0)	0.7 (0.0,1.0)	0.2 (0.1,0.6)	0.2 (0.0,0.9)	0.8 (0.1,1.0)	0.4 (0.0,0.8)
Post	7.5 (6.0,8.5)	0.6 (0.0,0.9)	0.8 (0.4,1.0)	0.4 (0.1,1.0)	0.0 (0.0,0.3)	0.1 (0.0,0.7)	0.2 (0.1,0.3)	0.9 (0.1,1.0)	0.2 (0.0,1.0)	0.8 (0.0,1.0)
7										
Pre	6.3 (4.0,8.5)	0.8 (0.2,1.0)	0.5 (0.3,0.7)	0.2 (0.0,0.7)	0.2 (0.0,0.5)	0.2 (0.0,0.7)	0.6 (0.4,1.0)	0.4 (0.2,0.6)	0.7 (0.3,0.9)	0.4 (0.1,0.9)
Post	6.0 (4.0,9.0)	0.7 (0.6,0.9)	0.5 (0.2,0.9)	0.4 (0.0,0.8)	0.2 (0.0,1.0)	0.1 (0.0,0.6)	0.4 (0.0,0.8)	0.5 (0.2,1.0)	0.5 (0.1,0.9)	0.5 (0.2,0.8)

PBC = perceived behavioural control

^a^Reporting: median (range). All questionnaire items were completed on a scale ranging from 0.0–1.0 except sleep length, which was completed on a scale of 0.0–12.0 and had an interval of 0.5 units. A higher score indicated higher rating (e.g. better sleep quality, more hours of sleep) apart from PBC, where a higher score indicated higher perceived difficulty (i.e. less perceived behavioural control) to engage in PA

**Table 4:** Descriptive statistics for the evening questionnaire data for each participant pre- and post-retirement^a^

Participant	PA facilitation	PA conflict	PA personalised^b^
1			
Pre	0.6 (0.0,0.8)	0.4 (0.2,0.8)	0.2 (0.0,0.6)
Post	0.7 (0.1,0.9)	0.3 (0.2,0.9)	0.0 (0.0,0.2)
2			
Pre	0.6 (0.0,1.0)	0.5 (0.0,1.0)	0.7 (0.1,1.0)
Post	0.7 (0.1,1.0)	0.5 (0.1,0.9)	0.7 (0.1,0.9)
3			
Pre	0.3 (0.0,1.0)	0.8 (0.0,1.0)	1.0 (0.2,1.0)
Post	0.3 (0.0,1.0)	0.8 (0.0,1.0)	1.0 (0.4,1.0)
4			
Pre	0.4 (0.0,1.0)	0.6 (0.0,1.0)	0.4 (0.0,1.0)
Post	0.5 (0.0,0.9)	0.2 (0.0,0.9)	0.3 (0.0,0.9)
5			
Pre	0.5 (0.2,0.9)	0.8 (0.2,1.0)	0.1 (0.0,0.9)
Post	0.6 (0.2,1.0)	0.4 (0.0,0.9)	0.7 (0.0,0.9)
6			
Pre	0.0 (0.0,0.8)	1.0 (0.3,1.0)	0.8 (0.1,1.0)
Post	0.3 (0.0,1.0)	0.5 (0.0,1.0)	0.6 (0.0,0.8)
7			
Pre	0.2 (0.1,0.8)	0.7 (0.2,1.0)	0.5 (0.0,1.0)
Post	0.3 (0.0,0.8)	0.5 (0.0,0.9)	0.2 (0.0,1.0)

^a^Reporting: median (range). These questions were asked at the end of the day in reference to that day

^b^Participant 1 answered ‘How much did your husband influence your PA today?’ Participant 2, 4, 6 and 7 answered ‘To what extent did other people influence your PA today?’ Participant 3 answered ‘How would you rate your asthma symptoms today?’ Participant 5 ‘How did seeing your partner today affect how much PA you did?’

### Dynamic regression results

The analysis was performed using awake data only. Table 5 describes the multivariable results for each participant (see Additional file 3 for detailed multivariable models for each participant).

**Table 5:** Overview table showing multivariable associations between predictor variables and PA bouts for all participants

		Participant 1	Participant 2	Participant 3	Participant 4	Participant 5	Participant 6	Participant 7
Variable								
*Main effects*								
Time (study day)		−0.004	−0.003***	0.000	0.001	0.004*	0.006	0.017*
Retirement^a^								
	Post-retirement	0.204	−0.410	0.057	1.471*	−0.115	0.567	0.801**
Day of week^b^								
	Weekend	−0.111	−0.027	−0.188***	0.140	−0.004	0.062	−0.021
Period of day^c^								
	Morning	−0.128	0.157**	0.145	−0.050	0.119	0.061	0.070
	Evening	−1.114***	−0.466***	−0.581***	−1.274***	−0.321***	−0.510***	−0.727***
Sleep length		0.127* (lag 0)	–	–	–	–	–	0.099* (lag 0)
		0.150** (lag 2)						
Sleep quality		−0.953* (lag 0)	–	–	–	0.372 (lag 1)	0.561** (lag 0)	–
Happiness		−1.035* (lag 2)	**–**	0.671* (lag 0)	1.200* (lag 0)	–	1.126** (lag 0)	–
				0.390* (lag 1)				
Tiredness		–	−0.475* (lag 0)	–	–	–	–	–
Stress		1.090* (lag 0)	–	0.655* (lag 0)	−0.282 (lag 1)	–	–	1.511** (lag 0)
				0.348* (lag 1)				
Time pressure		–	–	–	–	–	0.889*** (lag 0)	–
Pain		1.295 (lag 0)	–	–	–	–	–	–
PA intention		–	–	−0.434* (lag 0)	0.456** (lag 0)	–	1.201*** (lag 0)	−0.435 (lag 2)
					0.506** (lag 1)			
PA PBC		−0.802** (lag 2)	–	−0.712*** (lag 0)	–	–	–	–
				0.211* (lag 1)				
PA priority		–	–	–	−0.361* (lag 1)	–	–	−0.702* (lag 0)
PA facilitation		–	–	0.432* (lag 2)	0.635*** (lag 0)	–	–	0.548* (lag 0)
PA conflict		–	−0.720*** (lag 0)	0.444* (lag 2)	0.291* (lag 1)	−0.683*** (lag 0)	–	0.436** (lag 1)
						0.316* (lag 1)		
PA personalised		−1.051* (lag 0)	–	–	−0.263* (lag 0)	−0.442*** (lag 0)	–	−0.388** (lag 1)
		1.086* (lag 2)						−0.383** (lag 2)
PA bouts ^prior 2 h^		0.042	−0.063**	−0.039*	−0.016	−0.050*	0.019	−0.053
PA bouts ^per day^		0.007 (lag 1)	−0.009 (lag 1)	0.000 (lag 1)	0.002 (lag 1)	0.021** (lag 1)	−0.016 (lag 1)	0.018 (lag 0)
		−0.002 (lag 2)	0.013* (lag 2)	−0.004 (lag 2)	0.004 (lag 2)	0.003 (lag 2)	−0.015 (lag 2)	0.013 (lag 1)
***Interaction effects***								–
Retirement*Time (study day)		–	–	–	–	–	–	−0.018**
Retirement*Day of week		–	–	–	−0.291*	–	**–**	**–**
Retirement*Period of day^d^								
	Pre-retirement morning		–			–	–	–
	Pre-retirement evening	0.741**	–	0.291**	0.781***	–	–	–
	Post-retirement morning		–			–	–	–
	Post-retirement afternoon	−0.313	–	0.018	−0.250*	–	–	–
	Post-retirement evening		–			–	–	–
Retirement*Sleep quality		1.178*	0.840**	–	–	–	–	–
Retirement*Happiness		**–**	0.556	−0.855*	−1.368	–	–	–
Retirement*Tiredness		–	–	–	–	–	−0.539*	–
Retirement*Stress		−1.777**	–	−0.776*	–	–	–	−1.600**
Retirement*PA intention		–	–	0.949**	–	–	−1.582***	–
Retirement*PA PBC		–	−0.394*	0.779**	–	–	–	0.480*
Retirement*PA facilitation		0.821	–	–	–	–	0.680*	–
Retirement*PA conflict		–	–	–	–	–	0.603*	−0.597**

* = *p* < 0.05; ** = *p* < 0.01; *** *p* < 0.001

^a^Reference category: pre-retirement

^b^Reference category: work day

^c^Reference category: afternoon

^d^Reference category: afternoon pre-retirement

#### Does PA change from pre- to post-retirement?

There was a statistically significant increase in the probability of engaging in PA bouts for participants 4 and 7 pre- to post-retirement (*p* = 0.03 and *p* = 0.001), with participant 7 also showing an increase of PA over time (*p* = 0.017). Participant 2 showed a statistically significant time trend for decreased probability of engaging in PA bouts over the study period (*p* < 0.001) and participant 5 showed a statistically significant time trend for increased probability of engaging in PA bouts over the study period (*p* = 0.019).

#### What are the predictors of PA bouts?

Participant 3 was less likely to engage in PA bouts at weekends (*p* < 0.001). There was a decreased probability of engaging in PA bouts in the evening compared to the afternoon for all participants (all *p* < 0.001). For participant 2 there was an increased probability of engaging in PA bouts in the morning compared to the afternoon (*p* = 0.002). There was considerable variation between participants concerning measured PA determinants on PA bouts. There was no consistent pattern of PA predictors across participants; all predictor variables (except pain) were associated with PA bouts for at least one participant. For example, sleep length was only significantly associated with engaging in PA bouts for participants 1 and 7 (*p* = 0.022; *p* = 0.018, respectively) and sleep quality was only significantly associated with engaging in PA bouts for participant 1 and 6 (*p* = 0.045; *p* = 0.003, respectively). For participant 1, not only was there an increase in probability of engaging in PA bouts with more hours of sleep the previous night, but the amount of sleep accumulated 2 days before was also associated with an increase probability of engaging in PA bouts on the current day. The personalised questionnaire item was a significant predictor of PA bouts for participants 1, 4, 5 and 7. Participants 4 and 5 engaged in less PA bouts on days when they reported greater influence from their spouse/other people on their PA (*p* = 0.032 and *p* < 0.001, respectively). Participant 1 engaged in less PA bouts on days when they reported greater spousal influence on their PA and more PA bouts on days when they reported greater spousal influence 2 days prior (*p* = 0.012). Participant 7 engaged in less PA bouts when they reported greater influence from other people on their PA 2 days prior (*p* = 0.003) and 1 day prior (*p* = 0.005). For participant 2, 3 and 5, the number of PA bouts in the previous two hours was significantly associated with an increased probability of PA bouts (*p* = 0.002; *p* = 0.018; *p* = 0.039, respectively). For two participants, the number of PA bouts in the previous 2 days (participant 2) and previous day (participant 5) were significantly associated with an increased probability of PA bouts on the current day (*p* = 0.037; *p* = 0.009, respectively).

#### Is retirement interacting with the other predictors?

Although the results show that retirement did not have a statistically significant effect in the probability of engaging in PA for most participants, retirement interacted with some of the predictors for all participants, except participant 5. There was a significant interaction between period of the day and retirement status on PA bouts for participants 1, 3 and 4. In addition, higher ratings on the following variables were associated with an increased probability of PA bouts in the post-retirement period; sleep quality (participants 1 and 2; *p* = 0.042 and *p* = 0.004, respectively), PA intention (participant 3; *p* = 0.002), PA PBC (indicating more difficulty to engage in PA, participants 3 and 7; *p* = 0.001 and *p* = 0.029, respectively), PA facilitation (participant 6; *p* = 0.010) and PA conflict (participant 6; *p* = 0.049). Lower ratings on the following variables were associated with an increased probability of PA bouts in the post-retirement period; tiredness (participant 6; *p* = 0.040), happiness (participant 3; *p* = 0.017), stress (participants 1, 3 and 7; *p* = 0.004, *p* = 0.036 and *p* = 0.002, respectively), PA intention (participant 6; *p* < 0.001), PA PBC (indicating less difficulty to engage in PA, participant 2; *p* = 0.032) and PA conflict (participant 7; *p* = 0.002).

## Discussion

### Main findings

There was a significant change (i.e. increase) in the number of PA bouts performed after retirement for two of the seven participants in this study. There was no consistent pattern of PA predictors across participants; all predictor variables (except pain) were associated with PA bouts for at least one participant. However, none were associated with PA bouts for ***all*** participants. The predictors that influenced the probability of engaging in PA bouts the most for each participant were as follows: stress (participants 1 and 7); the extent to which other activities conflicted with engaging in PA (participants 2 and 5); PBC for engaging in PA (participant 3); happiness (participant 4) and intention to engage in PA (participant 6). For five participants, the relationship between PA determinants and PA bouts changed pre- to post-retirement.

### Relationship to previous research

For most participants, there was no significant change in the number of PA bouts performed pre- to post-retirement. The findings from this study are in contrast to existing evidence which show that PA levels change, either in a positive or negative direction, after retirement [11]. Previous studies have used research designs that focus on the change in PA after retirement on ***average*** in a population sample, which may have obscured individual trajectories. Due to the nature of aggregation the average PA trajectory after retirement may not represent all or any of the individuals studied [23]. Furthermore, previous studies have measured PA several years post-retirement when individuals are likely to have settled into a retirement routine. As a result, these studies provide limited information about PA around the transition to retirement. This information is needed to inform interventions targeting the retirement transition, when individuals may be more receptive to interventions targeting health behaviours [4, 15].

This study focused on the retirement *transition* and found that most individuals compensate for their pre-retirement PA in the period immediately after they retire. However, PA at this level may not be sustained over time beyond this period. This may explain why previous studies, which have measured PA from at least 6 months up to several years post-retirement [e.g. 9] have found changes in PA. Furthermore, most of the previous studies in this area have measured changes in PA via global questionnaires that involved retrospectively self-reporting PA over long periods of time. In contrast, this study used objective methods to measure day-to-day PA changes and focused specifically on short bouts of PA which have been linked to important health benefits [1]. Theories about how individuals adjust to retirement may provide insight into the heterogeneity of PA levels and determinants during the retirement transition. According to Atchley [34] individuals can enter one of three potential phases after leaving work: immediate retirement (i.e. a routine is established very quickly after retirement), the honeymoon phase (where individuals seek opportunities to adopt new activities and/or travel), and rest and relaxation (a period of rest after a busy career). Individuals entering retirement may experience one or more of these stages and the order in which they experience them may vary between individuals [34]. These different phases may have differential effects on PA levels and determinants after retirement [15].

This study identified the time of day as a predictor of PA. For the majority of the participants, there was significantly more PA bouts performed in the morning and fewer in the evening. This is consistent with previous research which has demonstrated that older adults engage in less PA in the evening [35]. A number of evidence- and theory-based PA determinants were measured as possible predictors of PA. There were no consistent predictors of PA across all individuals and this conflicts with evidence from group-based studies. For example, evidence from a large body of group-based studies suggests that intention and PBC are important determinants of PA behaviour [36]. However, this study found that these constructs do not predict the PA of all individuals. Previous studies using n-of-1 methods to identify intra-individual predictors of PA have also shown that group-based predictors of PA may not apply to individuals [24, 25]. Although there was no single variable that predicted PA for all participants in the study, the most consistent determinants of PA across participants were happiness, PA conflict and the personalised questionnaire items (which were related to the influence of other people for six of the seven participants). Mood has been identified as a predictor of PA in a number of studies [19]. Furthermore, the PA behaviour, attitude and supportive nature of a spouse is known to play an important role for PA behaviour in retirement [37].

The findings show that the relationship between PA and predictors may change pre- to post-retirement, despite no significant changes to the amount of PA bouts performed. This supports the view that the retirement transition may represent a ‘window’ of opportunity or ‘teachable moment’, when older adults may be prepared to change their PA in response to the reduction of common barriers to PA such as time availability, tiredness, stress and lack of motivation during this period [15].

### Strengths and limitations

This study is the first of its kind to use an n-of-1 natural experimental design to understand changes in PA over time during the retirement transition. A major strength of this study is the use of objective measurement of PA and the use of EMA [38, 39]. The majority of existing studies exploring PA change after retirement have used self-reported measures of PA [11]. EMA involved real-time sampling of cognitions and affect in the participant’s natural environment. This limits reliance on retrospective memory processes that can be biased by mood and situational factors [39]. This study is the first to apply dynamic regression modelling to evaluate changes in an individual’s health behaviour over time. The majority of n-of-1 studies conducted in health behaviour research have used visual analysis to evaluate n-of-1 data [23], which does not account for existing trends or autocorrelation within the data and can bias interpretations [40]. Dynamic modelling adjusts for autocorrelation by conditioning on the past. Unlike conventional autoregressive models, which only include the previous response directly in the model, dynamic modelling allows selection of the best function of the past. The appropriateness of the dynamic model, including the interpretation of the coefficients for the lagged variables, depends on some statistical assumptions [41]. Firstly, it is assumed that the effect of the past is captured through the exogenous, endogenous and dynamic covariates described. It is also assumed that the dynamics do not change over time and that the dynamic covariates do not lie on the causal pathways between the exogenous/endogenous covariates and the outcome. Finally, there was minimal missing accelerometer and questionnaire data. Reasons for high levels of compliance with the study procedures may be related to the fact that some aspects of the study design were personalised to each participant’s preferences and interests.

Some limitations are acknowledged. Due to the use of a natural experimental design involving a pre- and post-retirement phase, it is not known whether the observed PA changes for participants 4 and 7 were the result of retirement or another external factor occurring simultaneously [40]. Identified changes in PA and determinants after retirement may be related to other situational or seasonal factors. Using fixed-schedule EMA prompts may have encouraged changes in cognitions and behaviour around the time of the prompt due to participant expectation [42]. A simple imputation rule was used to impute missing questionnaire data and no imputation was carried out for missing accelerometer data. Imputing missing time series data can be problematic due to potential autocorrelation within the data. However, the minimal amount of missing data did not warrant the use of complex multiple imputation procedures [43].

### Implications for future research and practice

Future research should consider long-term objective measurement of PA to identify whether PA patterns remain stable or change over time beyond the retirement transition period. Older adults engage in a high level of sedentary behaviour, an independent health risk factor that can co-occur alongside high levels of PA [44]. Longitudinal group-based studies have shown that levels of sedentary behaviour can change after retirement [e.g. 6] but future research should explore whether group-level findings apply to individuals [45]. Two participants demonstrated a statistically significant change in PA post-retirement. The clinical significance of these findings should be considered.

There is significant diversity between individuals concerning why and when they retire, and a number of possible PA trajectories after retirement. Therefore, a ‘one-size-fits-all’ approach to intervention development is not appropriate and may explain why previous interventions that have specifically targeted retirees have not been successful [46, 47]. To account for the unique nature of retirement the most effective strategies to change health behaviours such as PA may be those that are highly personalised and meet the needs, preferences, roles and goals that individuals have when entering the retirement transition [15]. N-of-1 methods could be used in clinical practice as a tool for developing personalised interventions that target the unique determinants of PA. For example, sleep quantity was identified as an important predictor of PA bouts for participant 1 during an observational phase; therefore, this participant may benefit from an intervention which targets sleep alongside PA. Effective interventions may be those delivered before retirement but with the ability to adapt over time [48] to account for unique changes in PA determinants during the retirement transition.

## Conclusion

We found there to be a statistically significant change in PA bouts for two individuals during the retirement transition. PA determinants varied considerably between individuals and for some individuals the predictors changed pre- to post-retirement. N-of-1 methods can identify intra-individual PA trajectories and determinants over time. N-of-1 methods can be used as a tool to aid the development of personalised PA interventions for older adults in the retirement transition.

## Additional files

Additional file 1: Daily questionnaire items. (DOCX 15 kb)

Additional file 2: ‘Awake’ wear and non-wear hours using accelerometer data. (DOCX 16 kb)

Additional file 3: Overview tables showing multivariate associations between explanatory variables and PA bouts for each participant. (DOCX 48 kb)

## Abbreviations

PA: Physical activity
