# Supplementary material for: Changes in physical activity during the retirement transition: a series of novel n-of-1 natural experiments
Source: Int J Behav Nutr Phys Act. 2017 Dec 8;14:167. doi: 10.1186/s12966-017-0623-7 (PMC5723062; doi:10.1186/s12966-017-0623-7)
Supplement: Supplementary file 1 — Daily questionnaire items. (DOCX 15 kb) [file 12966_2017_623_MOESM1_ESM.docx]

Additional file 1. Daily questionnaire items^a^

| **Variable** | **Questionnaire item** | **Scale anchors** |
| --- | --- | --- |
| ***Morning questionnaire***^b^ |  |  |
| Sleep length | How many hours did you sleep last night? | 0.0-12.0 |
| Sleep quality | How would you rate the quality of your sleep? | very bad-very good |
| Happiness | Right now, to what extent are you happy? | not at all-very much |
| Tiredness | Right now, to what extent are you tired? | not at all-very much |
| Stress | Right now, to what extent are you stressed? | not at all-very much |
| Time pressure | Right now, to what extent are you under time pressure? | not at all-very much |
| Pain | Right now, to what extent are you in pain? | not at all-very much |
| Intention to engage in PA | How much do you intend to engage in PA today? | not at all-very much |
| PBC to engage in PA | How difficult is it for you to engage in PA today? | not at all-very much |
| Priority to engage in PA | How much of a priority is engaging in PA compared to doing the other things you have to do today? | not at all-very much |
| ***Evening questionnaire*** |  |  |
| PA facilitation | To what extent did the things you were doing today prevent you from engaging in PA? | not at all-very much |
| PA conflict | To what extent did the things you were doing today lead you to engage in PA? | not at all-very much |
| Personalised item | How much did your husband influence your PA today? (participant 1)  To what extent did other people influence your PA today? (participant 2)  How would you rate your asthma symptoms today? (participant 3)  To what extent did other people influence your PA today? (participant 4)  How did seeing your partner today affect how much PA you did? (participant 5)  To what extent did other people influence your PA today? (participant 6)  To what extent did other people influence your PA today? (participant 7) | not at all- very much  not at all- very much  very bad-very good  not at all- very much  not at all- very much  not at all- very much  not at all- very much |

PBC = perceived behavioural control

^a^ All questionnaire items were answered on a visual analogue scale of 0.00-1.00 except sleep length which was answered on a scale of 0.0-12.0 and had an interval of 0.5 units. A higher score indicated higher rating (e.g. better sleep quality, more hours of sleep, higher perceived difficulty [PBC] to engage in PA).

^b^ All morning questionnaire items were ecological momentary assessments with the exception of sleep length and sleep quality
