# Supplementary material for: Changes in physical activity during the retirement transition: a series of novel n-of-1 natural experiments
Source: Int J Behav Nutr Phys Act. 2017 Dec 8;14:167. doi: 10.1186/s12966-017-0623-7 (PMC5723062; doi:10.1186/s12966-017-0623-7)
Supplement: Supplementary file 2 — ‘Awake’ wear and non-wear hours using accelerometer data. (DOCX 16 kb) [file 12966_2017_623_MOESM2_ESM.docx]

Additional file 2. ‘Awake’ wear and non-wear hours using accelerometer data

| Participant |  | No. days in study | No. of awake hours | | No. of awake non-wear hours  (missing data) | |
| --- | --- | --- | --- | --- | --- | --- |
|  |  |  | Per day^1^ | Total^2^ | Per day^1^ | Total^2^ |
| **1** |  |  |  |  |  |  |
| Pre-retirement  Post-retirement  Total | | 43 (35.5)  78 (64.5)  121 | 14.7 (12.5,16.6)  13.9 (10.9,16.8)  14.2 (10.9,16.8) | 629 (36.2)  1107 (63.8)  1736 (100) | 0 (0,0)  0 (0,0)  0 (0,0) | 0 (0.0)  0 (0.0)  0 (0.0) |
| **2** | |  |  |  |  |  |
| Pre-retirement  Post-retirement  Total | | 72 (38.1)  117 (61.9)  189 | 13.7 (10.6,15.6)  13.5 (9.2,15.6)  13.6 (9.2,15.6) | 937 (37.2)  1548 (62.8)  2521(97.2) | 0 (0,3)  0 (0,4)  0 (0,4) | 28 (38.9)  44 (61.1)  72 (2.8) |
| **3** |  |  |  |  |  |  |
| Pre-retirement  Post-retirement  Total | | 81 (41.3)  115 (58.7)  196 | 16.4 (12.6,18.1)  16.1 (11.7,19.2)  16.3 (11.7,19.2) | 1239 (40.1)  1854 (59.9)  3093 (99.5) | 0 (0,2)  0 (0,2)  0 (0,2) | 7 (43.8)  9 (56.3)  16 (0.5) |
| **4** |  |  |  |  |  |  |
| Pre-retirement  Post-retirement  Total | | 91 (46.4)  105 (53.6)  196 | 15.5 (11.1,17.8)  14.9 (12.8,16.7)  15.1 (11.1,17.8) | 1391 (47.0)  1568 (53.0)  2959 (99.8) | 0 (0,0)  0 (0,3)  0 (0,3) | 0 (0.0)  6 (100)  6 (0.2) |
| **5** |  |  |  |  |  |  |
| Pre-retirement  Post-retirement  Total | | 43 (34.4)  82 (65.6)  125 | 14.9 (6.6,16.9)  13.7 (6.7, 16.0)  14.0 (6.6,16.9) | 625 (36.5)  1084 (63.5)  1710 (95.7) | 0 (0,7)  0 (0,7)  0 (0,7) | 13 (17.1)  63 (82.9)  76 (4.2) |
| **6** |  |  |  |  |  |  |
| Pre-retirement  Post-retirement  Total | | 33 (37.9)  54 (62.1)  87 | 14.6 (11.5,16.4)  14.1 (6.3,16.2)  14.2 (6.3,16.4) | 472 (39.6)  719 (60.4)  1191 (97.9) | 0 (0,3)  0 (0,8)  0 (0,8) | 7 (26.9)  19 (73.1)  26 (2.1) |
| **7** |  |  |  |  |  |  |
| Pre-retirement  Post-retirement  Total | | 36 (20.3)  141 (79.7)  177 | 16.1 (12.6,18.3)  16.2 (10.5,19.3)  16.1 (10.5,19.3) | 573 (18.9)  2268 (79.8)  2842 (96.7) | 0 (0,4)  0 (0,7)  0 (0,7) | 19 (19.4)  79 (80.6)  98 (3.3) |

^1^ Reporting: median (range)

^2^ Reporting: n (%)
