# Supplementary material for: Changes in physical activity during the retirement transition: a series of novel n-of-1 natural experiments
Source: Int J Behav Nutr Phys Act. 2017 Dec 8;14:167. doi: 10.1186/s12966-017-0623-7 (PMC5723062; doi:10.1186/s12966-017-0623-7)
Supplement: Supplementary file 3 — Overview tables showing multivariate associations between explanatory variables and PA bouts for each participant. (DOCX 48 kb) [file 12966_2017_623_MOESM3_ESM.docx]

Additional file 3. Overview tables showing multivariate associations between explanatory variables and PA bouts for each participant.

Participant 1

| **Variable name** | **β** | **SE** | **z** | ***p*** |
| --- | --- | --- | --- | --- |
| Time (study day) | -0.004 | 0.003 | -1.729 | 0.084 |
| Retirement^1^  Post-retirement | 0.204 | 0.424 | 0.482 | 0.630 |
| Day of week^2^  Weekend | -0.111 | 0.111 | -1.000 | 0.317 |
| Sleep length (lag 0) | 0.127 | 0.055 | 2.286 | 0.022 |
| Sleep quality (lag 0) | -0.953 | 0.475 | -2.008 | 0.045 |
| Stress (lag 0) | 1.090 | 0.431 | 2.527 | 0.012 |
| Pain (lag 0) | 1.295 | 0.662 | 1.955 | 0.051 |
| PA personalised item^3^ (lag 0) | -1.051 | 0.502 | -2.094 | 0.036 |
| Period of day^4^  Morning  Evening | -0.128  -1.114 | 0.167  0.227 | -0.769  -4.900 | 0.442  <0.001 |
| PA bouts ^per day^ (lag 1) | 0.007 | 0.016 | 0.423 | 0.672 |
| PA bouts ^per day^ (lag 2) | -0.002 | 0.016 | -0.105 | 0.916 |
| Sleep length (lag 2) | 0.150 | 0.051 | 2.912 | 0.004 |
| Happiness (lag 2) | -1.035 | 0.472 | -2.191 | 0.028 |
| PA PBC (lag 2) | -0.802 | 0.280 | -2.867 | 0.004 |
| PA personalised item^3^ (lag 2) | 1.086 | 0.434 | 2.500 | 0.012 |
| PA bouts ^prior 2 hours^ | 0.042 | 0.043 | 0.968 | 0.333 |
| Retirement x Period of day^5, 6^  Pre-retirement morning  Pre-retirement evening  Post-retirement morning  Post-retirement afternoon  Post-retirement evening | NA  0.741  NA  -0.313  NA | NA  0.254  NA  0.199  NA | NA  2.914  NA  -1.572  NA | NA  0.004  NA  0.116  NA |
| Retirement x Sleep quality^5^ | 1.178 | 0.579 | 2.036 | 0.042 |
| Retirement x Stress^5^ | -1.777 | 0.623 | -2.851 | 0.004 |
| Retirement x PA facilitation^5^ | 0.821 | 0.422 | 1.944 | 0.052 |
|  |  |  |  |  |
| Goodness of fit: χ^2^ = 4.078, *p* = 0.850 | | | | |

^1^Reference category: pre-retirement

^2^Reference category: work day

^3^‘How much did your husband influence your PA today?’

^4^Reference category: afternoon

^5^Interaction

^6^Reference category: afternoon pre-retirement

NA = not available. The variable is linearly related to some other variables in the model

PBC = Perceived behavioural control

Participant 2

| **Variable name** | **β** | **SE** | **z** | ***P*** |
| --- | --- | --- | --- | --- |
| Time (study day) | -0.003 | 0.001 | -3.667 | <0.001 |
| Retirement^1^  Post-retirement | -0.410 | 0.259 | -1.585 | 0.113 |
| Day of week^2^  Weekend | -0.027 | 0.054 | -0.493 | 0.622 |
| Period of day^3^  Morning  Evening | 0.157  -0.466 | 0.051  0.066 | 3.064  -7.097 | 0.002  <0.001 |
| PA bouts ^prior 2 hours^ | -0.063 | 0.021 | -3.054 | 0.002 |
| PA bouts ^per day^ (lag 1) | -0.009 | 0.006 | -1.424 | 0.154 |
| PA bouts ^per day^ (lag 2) | 0.013 | 0.006 | 2.081 | 0.037 |
| PA conflict (lag 0) | -0.720 | 0.106 | -6.791 | <0.001 |
| Retirement x PA PBC^4^ | -0.394 | 0.184 | -2.142 | 0.032 |
| Retirement x Happiness^4^ | 0.556 | 0.288 | 1.930 | 0.054 |
| Retirement x Sleep quality^4^ | 0.840 | 0.294 | 2.851 | 0.004 |
| Tired (lag 0) | -0.475 | 0.228 | -2.081 | 0.037 |
|  |  |  |  |  |
| Goodness of fit: χ^2^ = 7.366, *p* = 0.498 | | | | |

^1^Reference category: pre-retirement

^2^Reference category: work day

^3^Reference category: afternoon

^4^Interaction

PBC = Perceived behavioural control

Participant 3

| **Variable name** | **β** | **SE** | **z** | ***P*** |
| --- | --- | --- | --- | --- |
| Time (study day) | 0.000 | 0.001 | 0.144 | 0.886 |
| Retirement^1^  Post-retirement | 0.057 | 0.428 | 0.132 | 0.895 |
| Day of week^2^  Weekend | -0.188 | 0.052 | -3.611 | <0.001 |
| Happiness (lag 0) | 0.671 | 0.279 | 2.403 | 0.016 |
| Stress (lag 0) | 0.655 | 0.305 | 2.145 | 0.032 |
| PA intention (lag 0) | -0.434 | 0.214 | -2.028 | 0.043 |
| PA PBC (lag 0) | -0.712 | 0.185 | -3.859 | 0.000 |
| Period of day^3^  Morning  Evening | 0.145  -0.581 | 0.076  0.103 | 1.919  -5.643 | 0.055  <0.001 |
| PA bouts ^per day^ (lag 1) | 0.000 | 0.005 | -0.013 | 0.990 |
| PA bouts ^per day^ (lag 2) | -0.004 | 0.005 | -0.814 | 0.416 |
| Happiness (lag 1) | 0.390 | 0.181 | 2.162 | 0.031 |
| Stress (lag 1) | 0.348 | 0.156 | 2.231 | 0.026 |
| PA PBC (lag 1) | 0.211 | 0.082 | 2.566 | 0.010 |
| PA facilitation (lag 2) | 0.432 | 0.189 | 2.286 | 0.022 |
| PA conflict (lag 2) | 0.444 | 0.173 | 2.563 | 0.010 |
| PA bouts ^prior 2 hours^ | -0.039 | 0.017 | -2.363 | 0.018 |
| Retirement x Period of day^4, 5^  Pre-retirement morning  Pre-retirement evening  Post-retirement morning  Post-retirement afternoon  Post-retirement evening | NA  0.291  NA  0.018  NA | NA  0.110  NA  0.094  NA | NA  2.650  NA  0.190  NA | NA  0.008  NA  0.849  NA |
| Retirement x Happiness | -0.855 | 0.358 | -2.390 | 0.017 |
| Retirement x Stress | -0.776 | 0.370 | -2.099 | 0.036 |
| Retirement x PA intention | 0.949 | 0.313 | 3.028 | 0.002 |
| Retirement x PA PBC | 0.779 | 0.245 | 3.176 | 0.001 |
|  |  |  |  |  |
| Goodness of fit: χ^2^ = 8.479, *p* = 0.388 | | | | |

^1^Reference category: pre-retirement

^2^Reference category: work day

^3^Reference category: afternoon

^4^Interaction

^5^Reference category: afternoon pre-retirement

NA = not available. The variable is linearly related to some other variables in the model

PBC = Perceived behavioural control

Participant 4

| **Variable name** | **β** | **SE** | **Z** | ***P*** |
| --- | --- | --- | --- | --- |
| Time (study day) | 0.001 | 0.001 | 0.924 | 0.355 |
| Retirement^1^  Post-retirement | 1.471 | 0.685 | 2.147 | 0.032 |
| Day of week^2^  Weekend | 0.140 | 0.091 | 1.548 | 0.122 |
| Happiness (lag 0) | 1.200 | 0.548 | 2.190 | 0.029 |
| PA intention (lag 0) | 0.456 | 0.156 | 2.919 | 0.004 |
| PA facilitation (lag 0) | 0.635 | 0.139 | 4.564 | <0.001 |
| PA personalised^3^ (lag 0) | -0.263 | 0.123 | -2.142 | 0.032 |
| Period of day^4^  Morning  Evening | -0.050  -1.274 | 0.090  0.140 | -0.555  -9.102 | 0.579  <0.001 |
| PA bouts ^per day^ (lag 1) | 0.002 | 0.009 | 0.205 | 0.838 |
| PA bouts ^per day^ (lag 2) | 0.004 | 0.008 | 0.489 | 0.625 |
| Stress (lag 1) | -0.282 | 0.162 | -1.739 | 0.082 |
| PA intention (lag 1) | 0.506 | 0.180 | 2.816 | 0.005 |
| PA priority (lag 1) | -0.361 | 0.149 | -2.447 | 0.014 |
| PA conflict (lag 1) | 0.291 | 0.135 | 2.152 | 0.031 |
| PA bouts ^prior 2 hours^ | -0.016 | 0.024 | -0.649 | 0.516 |
| Retirement x Day of week^5^ | -0.291 | 0.118 | -2.458 | 0.014 |
| Retirement x Period of day^5,6^  Pre-retirement morning  Pre-retirement evening  Post-retirement morning  Post-retirement afternoon  Post-retirement evening | NA  0.781  NA  -0.250  NA | NA  0.153  NA  0.116  NA | NA  5.105  NA  -2.162  NA | NA  <0.001  NA  0.031  NA |
| Retirement x Happiness | -1.368 | 0.784 | -1.745 | 0.081 |
|  |  |  |  |  |
| Goodness of fit: χ^2^ = 26.407, *p* = 0.001 | | | | |

^1^Reference category: pre-retirement

^2^Reference category: work day

^3^’To what extent did other people influence your PA today?’

^4^Reference category: afternoon

^5^Interaction

^6^Reference category: afternoon pre-retirement

NA = not available. The variable is linearly related to some other variables in the model

Participant 5

| **Variable name** | **β** | **SE** | **z** | ***p*** |
| --- | --- | --- | --- | --- |
| Time (study day) | 0.004 | 0.002 | 2.337 | 0.019 |
| Retirement^1^ | -0.115 | 0.112 | -1.020 | 0.308 |
| Day of week^2^  Weekend | -0.004 | 0.065 | -0.062 | 0.951 |
| Period of day^3^  Morning  Evening | 0.119  -0.321 | 0.066  0.069 | 1.793  -4.633 | 0.073  <0.001 |
| PA bouts ^prior 2 hours^ | -0.050 | 0.024 | -2.062 | 0.039 |
| PA bouts ^per day^ (lag 1) | 0.021 | 0.008 | 2.607 | 0.009 |
| PA bouts ^per day^ (lag 2) | 0.003 | 0.008 | 0.334 | 0.738 |
| PA conflict (lag 0) | -0.683 | 0.152 | -4.490 | <0.001 |
| PA personalised^4^ (lag 0) | -0.442 | 0.119 | -3.707 | <0.001 |
| PA conflict (lag 1) | 0.316 | 0.139 | 2.266 | 0.023 |
| Sleep quality (lag 1) | 0.372 | 0.201 | 1.848 | 0.065 |
|  |  |  |  |  |
| Goodness of fit: χ^2^ = 11.092, *p* = 0.197 | | | | |

^1^Reference category: pre-retirement

^2^Reference category: work day

^3^Reference category: afternoon

^4^’How did seeing your partner today affect how much PA you did?’

Participant 6

| **Variable name** | **β** | **SE** | **z** | ***p*** |
| --- | --- | --- | --- | --- |
| Time (study day) | 0.006 | 0.003 | 1.617 | 0.106 |
| Retirement^1^  Pre-retirement | 0.567 | 0.421 | 1.346 | 0.178 |
| Day of week^2^  Weekend | 0.062 | 0.095 | 0.657 | 0.511 |
| Sleep quality (lag 0) | 0.561 | 0.188 | 2.985 | 0.003 |
| Happiness (lag 0) | 1.126 | 0.363 | 3.098 | 0.002 |
| Time pressure (lag 0) | 0.889 | 0.247 | 3.596 | <0.000 |
| PA intention (lag 0) | 1.201 | 0.339 | 3.544 | <0.001 |
| Period of day^3^  Morning  Evening | 0.061  -0.510 | 0.096  0.093 | 0.633  -5.474 | 0.527  <0.001 |
| PA bouts ^per day^ (lag 1) | -0.016 | 0.013 | -1.265 | 0.206 |
| PA bouts ^per day^ (lag 2) | -0.015 | 0.014 | -1.076 | 0.282 |
| PA bouts ^prior 2 hours^ | 0.019 | 0.331 | 0.561 | 0.575 |
| Retirement x Tired^4^ | -0.539 | 0.262 | -2.056 | 0.040 |
| Retirement x PA intention^4^ | -1.582 | 0.385 | -4.111 | <0.001 |
| Retirement x PA facilitation^4^ | 0.680 | 0.264 | 2.570 | 0.010 |
| Retirement x PA conflict^4^ | 0.603 | 0.306 | 1.970 | 0.049 |
|  |  |  |  |  |
| Goodness of fit: χ^2^ = 6.644, *p* = 0.576 | | | | |

^1^Reference category: pre-retirement

^2^Reference category: work day

^3^Reference category: afternoon

^4^Interaction

Participant 7

| **Variable name** | **β** | **SE** | **z** | ***p*** |
| --- | --- | --- | --- | --- |
| Time (study day) | 0.017 | 0.007 | 2.387 | 0.017 |
| Retirement^1^  Post-retirement | 0.801 | 0.248 | 3.225 | 0.001 |
| Day of week^2^  Weekend | -0.021 | 0.069 | -0.302 | 0.763 |
| Sleep length (lag 0) | 0.099 | 0.042 | 2.376 | 0.018 |
| Stress (lag 0) | 1.511 | 0.460 | 3.282 | 0.001 |
| PA priority (lag 0) | -0.702 | 0.325 | -2.159 | 0.031 |
| PA facilitation (lag 0) | 0.548 | 0.251 | 2.186 | 0.029 |
| Period of day^3^  Morning  Evening | 0.070  -0.727 | 0.064  0.081 | 1.093  -8.942 | 0.274  <0.001 |
| PA bouts ^per day^ (lag 1) | 0.018 | 0.011 | 1.590 | 0.112 |
| PA bouts ^per day^ (lag 2) | 0.013 | 0.010 | 1.311 | 0.190 |
| PA conflict (lag 1) | 0.436 | 0.159 | 2.736 | 0.006 |
| PA personalised^4^ (lag 1) | -0.388 | 0.139 | -2.787 | 0.005 |
| PA intention (lag 2) | -0.435 | 0.240 | -1.816 | 0.069 |
| PA personalised^4^ (lag 2) | -0.383 | 0.127 | -3.019 | 0.003 |
| PA bouts ^prior 2 hours^ | -0.053 | 0.031 | -1.713 | 0.087 |
| Retirement x Time (study day)^5^ | -0.018 | 0.007 | -2.681 | 0.007 |
| Retirement x Stress^5^ | -1.600 | 0.523 | -3.059 | 0.002 |
| Retirement x PBC^5^ | 0.480 | 0.220 | 2.185 | 0.029 |
| Retirement x PA conflict^5^ | -0.597 | 0.195 | -3.070 | 0.002 |
|  |  |  |  |  |
| Goodness of fit: χ^2^ = 7.850, *p* = 0.448 | | | | |

^1^Reference category: pre-retirement

^2^Reference category: work day

^3^Reference category: afternoon

^4^‘To what extent did other people influence your PA today?’

^5^Interaction

PBC = Perceived behavioural control
